# Physical Activity Amount and Cognitive Impairment in Korean Elderly Population

**DOI:** 10.3390/brainsci10110804

**Published:** 2020-10-31

**Authors:** Seung-Taek Lim, Yung Zoon Jung, Takao Akama, Eunjae Lee

**Affiliations:** 1 Institute of Sport Science, Kangwon National University, Gangwon-do, Chuncheon-si 24341, Korea; limdotor@gmail.com; 2 Waseda Institute for Sport Sciences, Waseda University, Saitama 341-0018, Japan; 3 Institute for Bio-Health Integration of Medicine and Korean Medicine, Nasaret International Hospital, Incheon 21972, Korea; 4 Director of Laboratory Medicine, Nasaret International Hospital, Incheon 21972, Korea; j31195138@gmail.com; 5 Faculty of Sport Sciences, Waseda University, Saitama 341-0018, Japan; takao-akama@waseda.jp; 6 Center for Sport Science in Incheon, Incheon 22234, Korea

**Keywords:** physical activity, cognitive, elderly, hemoglobin, creatinine

## Abstract

The relationship between physical activity amount and cognitive function in elderly Koreans has received little attention. This study therefore aimed to understand the independent and common link between cognitive function and physical activity levels among elderly Korean adults. This study recruited a total of 2746 elderly adults (1348 males and 1398 females). All participants were assessed for cognitive functioning using the Korean Dementia Screening Questionnaire Cognition (KDSQ-C). The computerized Korean version short form of the International Physical Activity Questionnaire (IPAQ) used in this study was entirely based on the long, self-administered, usual week-long IPAQ found in the IPAQ manual of operation. In the unadjusted model, elderly adults who met the recommended level of moderate-to-vigorous physical activity were more likely to have a sufficient level of cognitive function. Hemoglobin, creatinine, cholesterol (TC), triglycerides (TG), LDL-C, and HDL-C differed significantly between groups. A negative correlation was found between KDSQ-C score and the moderate-to-vigorous physical activity (MVPA) amount per week. Physical activity amount is associated with cognition function in Korean elderly adults. Increasing physical activity may improve hemoglobin and creatinine and be involved in improving serum lipid profiles in elderly adults. Thus, physical activity has been suggesting as a useful tool to reduce the risk of cognitive function associated with aging.

## 1. Introduction

Due to a super-aged population, weakness has become an increasingly important issue in Korea [1]. In a study among South Korean women, *The Lancet* reported a 90% probability that life expectancy at birth in 2030 would exceed 86.7 years of age, which matches the highest worldwide life expectancy rate from 2012, and a 57% probability of exceeding 90 years of age [2].

Public health problems are increasing worldwide. This includes cognitive impairment, which is prevalent among the elderly at 10% to 22% [3]. Cognitive decline is a common part of aging that may instill fear [4]. For this reason, there is continual research into whether the condition indicates the early stages of dementia or is a part of a healthy continuum [5]. There are also a variety of effective preventive strategies that majorly impact public health by improving the quality of life while reducing associated economic and social burdens [6].

In the context of a substantially aging world population, many studies are now focusing on the potential positive effects of physical activity on cognitive impairment [7]. Current global guidelines for using physical activity to improve health encourage older adults to perform moderate-to-vigorous physical activity (MVPA) for at least 150 min each week [8,9]. Previous studies have also shown that endurance exercises can protect against cognitive decline among the elderly, especially in regard to elements of executive planning and the working memory [10], while aerobic exercise has been found to increase brain volume in the frontal region, thereby resulting in higher levels of processing, attention control, and working memory [11]. In this regard, regular exercise and other forms of physical activity are highly important for promoting cognitive function and health not only in the cognitively impaired, but also in the healthy elderly.

Additionally, low hemoglobin [12], low creatinine [13], low high-density lipoprotein cholesterol (HDL-C), and high triglycerides (TG) [14] are potential contributing factors for progressive cognitive decline in the elderly. These biomarkers are associated with vascular risk factors [15], leading to vascular dementia in elderly people [16]. Moreover, a lower hemoglobin level was independently associated with mortality in this elderly cohort study [17]. The low creatinine might simultaneously reflect decreased muscle mass in elderly populations [13].

Although previous studies have investigated cognitive impairment in the elderly, there has been relatively little focus on the relationship between the amount of physical activity and cognitive function in elderly Korean adults. This study therefore aimed to understand the independent and common link between cognitive function and physical activity levels among elderly Korean adults.

## 2. Methodology

### 2.1. Participants

This study recruited a total of 2746 elderly adults (1348 males and 1398 females). All participants were 65 years of age or older and were specifically recruited from Nasaret International Hospital (Incheon, South Korea) from January 2018 to December 2019.

All participants were screened for cognitive function using the Korean Dementia Screening Questionnaire Cognition (KDSQ-C) from the Ministry of Health and Welfare [18]. According to a baseline KDSQ-C cutoff score of 6 points, participants were divided into two groups, including the cognitive impairment group (scores ≥ 6 points) and normal group (scores < 6 points).

The data set was drawn from a retrospective cohort based on Nasaret International Hospital Medical Informatics Data (NIHMID), and separate patient recruitment procedures were not carried out. As the data were de-identified, the informed consent of the subject was not applicable. In the NIHMID, de-identified join keys replacing personal identifiers are used to secure ethical clearance. Therefore, the researcher cannot receive informed consent from individual patients for the use of personal information. However, the use of NIHMID for research purposes requires approval from the institutional review board. This study was approved by the Institutional Review Board at Kangwon National University (KWNUIRB-2019-07-009-003).

The characteristics of the participants are shown in Table 1.

### 2.2. Korean Dementia Screening Questionnaire Cognition (KDSQ-C)

As mentioned in the previous section, all participants were assessed for cognitive function using the Korean Dementia Screening Questionnaire Cognition (KDSQ-C) [18]. The KDSQ-C is self-administered and consists of 15 cognitive dysfunction items, each of which are rated on a three-point scale: 0 (no), 1 (sometimes/occasional), and 2 (often/frequent). The KDSQ-C is not influenced by age or educational level and has previously shown scores of 0.79 for sensitivity and 0.80~0.86 for specificity among dementia [19,20]. According to a baseline KDSQ-C cutoff score of 6 points, participants were divided into two groups, including the cognitive impairment group (scores ≥ 6 points) and normal group (scores < 6 points).

### 2.3. The International Physical Activity Questionnaire (IPAQ)

This study used the computerized Korean version of the IPAQ, which is entirely based on the long, self-administered, usual week-long IPAQ found in the IPAQ manual of operation. The 7-item IPAQ identifies the total minutes over the previous seven days that were spent on moderate-to-vigorous physical activity (MVPA), walking physical activity, and inactivity [21]. It is specifically designed to collect information on the amounts of time (i.e., the number of sessions and average amounts of time per session) spent walking, in moderate-intensity physical activity, in vigorous-intensity physical activity, and sitting on weekdays and weekend days. Questions regarding participation in moderate and vigorous physical activities were supplemented by concrete examples of commonly performed activities. Data obtained via the questionnaires were then summed for each item (i.e., vigorous intensity, moderate intensity, and walking) in order to estimate the total amount of time spent in physical activity on a weekly basis.

Based on the self-reported amount of time spent in MVPA, participants were categorized as either sufficiently or insufficiently active. This was done according to guidelines set forth by the American College of Sports Medicine (ACSM)/Centers for Disease Control and Prevention (CDC) [9] stating that individuals should accumulate at least 150 min of moderate-intensity activity per week.

### 2.4. Blood Collection and Hemoglobin, Creatinine, and Serum Lipid Analysis

Fasting venous blood samples were collected from all participants. Fasting was maintained for nine hours. Blood samples were collected on the following day. Participants were instructed to obtain sufficient sleep and refrain from radical movements as much as possible. Samples were immediately centrifuged at 3500× *g* at 4 °C for 10 min and analyzed within 24 h. Serum levels of total cholesterol (TC), triglycerides (TG), high-density lipoprotein cholesterol (HDL-C), low-density lipoprotein cholesterol (LDL-C), and creatinine were measured using a biochemical automatic analyzer using commercial kits (Hitachi 7180, Tokyo, Japan). Hemoglobin levels were determined using a hemoglobin assay kit (Sysmex XN-1000, Kobe, Japan) according to the manufacturer’s protocol.

### 2.5. Statistical Analysis

All statistical analyses were conducted using the SPSS statistical package for Windows, version 25.0 (SPSS, Inc., Chicago, IL, USA). Means and standard deviations were computed for all variables. Binary logistic regression analyses were also conducted to examine the independent and joint associations of sufficient MVPA levels with cognitive function; odds ratios (ORs) and 95% confidence intervals (CIs) were calculated for these associations. The participant group consisting of individuals who met the recommended MVPA levels (150 min per week) was set as the reference during the joint association analyses. The aforementioned covariates were adjusted for in the adjusted models. The relationships between KDSQ-C scores and weekly MVPA amounts were analyzed using Pearson’s correlation coefficients. The participant’s characteristics (age, height, weight, body mass index, waist circumference, systolic and diastolic blood pressure) and blood variables (hemoglobin, creatinine, TC, TG, LDL-C, and HDL-C) were further analyzed for significant difference among the groups using a one-way ANOVA. Finally, a post-hoc analysis (Bonferroni) was used to compare specific differences in cases of significance, which was statistically accepted at the 0.05 level.

## 3. Results

### 3.1. Associations between Physical Activity and Cognitive Function

Table 2 shows the independent associations between physical activity amounts and cognitive function in elderly adult participants. In the unadjusted model, participants who met the recommended MVPA levels (OR = 1.63, 95% CI = 1.28–2.08) were more likely to have a sufficient level of cognitive functioning. Those not meeting the recommended MVPA levels were 1.63 fold more likely to have cognitive decline. Additionally, the associations between meeting the recommended MVPA levels (OR = 1.35, 95% CI = 1.05–1.4) were attenuated after adjusting (age, sex, body mass index, and waist circumference) for covariates. Even with adjusting, elderly Korean adults who did not meet the recommended MVPA levels were 1.35 times more likely to have cognitive decline.

### 3.2. Hemoglobin, Creatinine, and Serum Lipids

Table 3 presents the hemoglobin, creatinine, and serum lipid profiles. A one-way ANOVA showed that hemoglobin (*p* < 0.001), creatinine (*p* < 0.001), TC (*p* < 0.001), TG (*p* = 0.018), LDL-C (*p* = 0.002), and HDL-C (*p* = 0.001) were significantly different between groups.

A post-hoc analysis using the Bonferroni test indicated that hemoglobin and creatinine levels in both the normal elderly and ≥150 min groups were significantly higher than those in the <150 min group (normal and cognitive impairment). Next, TC and LDL-C in the normal elderly and ≥150 min groups were significantly lower than those in the normal elderly and <150 min groups as well as in the cognitive impairment and ≥150 min groups.

### 3.3. Correlations Coefficients between KDSQ-C Scores and MVPA Amounts

**Figure 1:**
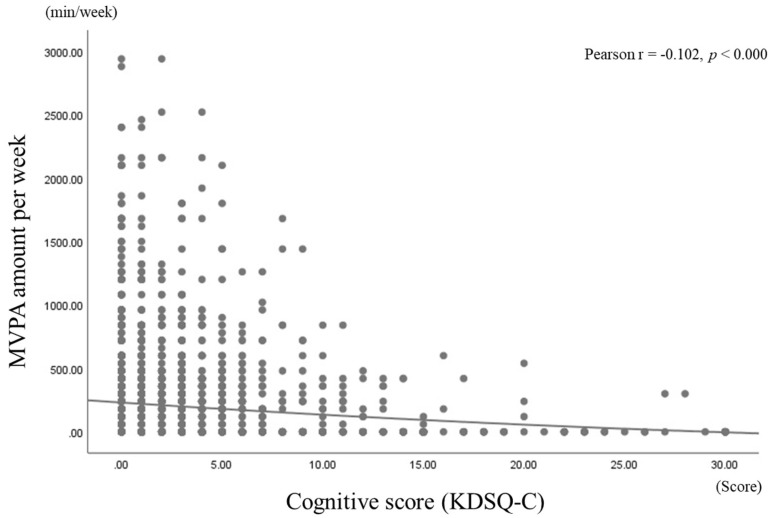
Pearson’s correlation coefficients for cognitive scores and weekly MVPA amounts.

Figure 1 shows the correlation coefficients between KDSQ-C scores and weekly MVPA amounts. As shown, a negative correlation was found between KDSQ-C scores and weekly MVPA amounts (*p* < 0.000).

## 4. Discussion

This study found that elderly Korean adults who did not engage in recommended MVPA levels exhibited approximately double the amount of decline in cognitive function when compared to those who met the recommendations. There was a negative correlation in which KDSQ-C scores increased as weekly MVPA amounts decreased. In addition, hemoglobin and creatinine levels were higher in the ≥150 min group than in the <150 min group, while TC and LDL-C were lower in the ≥150 min group than in the <150 min group.

Most clinical practice guidelines recommend screening to identify these patients at an early time in the primary care setting [22]. Research has shown that cognitive impairments are associated with decreased hippocampal capacity and reduced processing speed in both mild cognitive impairment and Alzheimer’s disease (AD) groups [23]. Further, research into muscle mass and other possible confounding factors has shown that participants with cognitive impairment have significantly diminished physical function when compared to those without cognitive impairment [24]. In addition, low upper and lower extremity performance levels are associated with increased disorder and dysfunction in the range of motion, even after considering the impacts of cognitive performance; this supports the suggestion that motor performance contributes to functional impairments in AD patients [25]. We produced strong evidence showing that elderly Korean adults who did not engage in recommended MVPA levels exhibited approximately double the amount of decline to cognitive function when compared to those who met the recommendations. In addition, we found a negative correlation in which KDSQ-C scores increased as weekly MVPA amounts decreased. Muscle strength is known to decrease in the elderly due to skeletal muscle atrophy [26]. As a result of aging, all body systems are inevitably reduced, thus leading to “weakness, fatigue, and slowing of movement” [27]. This shows that regular physical activity is essential, especially among the aging. Indeed, a substantial amount of evidence has been reported on cognitive function in this regard. For instance, a large sample size (*n* = 4615) of elderly Canadian women showed an association between lower cognitive loss risk and regular physical activity [28]. Further, a study among 732 healthy Greek adults aged 60 years or older at baseline (after about 6–13 years) showed a strong positive association between physical activity and Mini-Mental State Examination (MMSE) scores upon registration; this relationship supports the protective role of physical activity against cognitive impairment [29]. In this study, 150 min of MVPA per week was set as a cut-off following previous studies conducted by the WHO [8] and Pate et al. [9]. Korea has instituted a periodic general national health examination program that screens for cognitive impairment using the KDSQ-C [19]. A previous study examined questionnaire scores, finding that the area under the curve for the KDSQ-C was 0.75 for diagnosing dementia [30]. This study also used the KDSQ-C questionnaire to assess cognitive function.

We also found noticeably significant differences in which hemoglobin and creatinine levels in the normal elderly and ≥150 min groups were higher than those in the <150 min group (normal and cognitive impairment). As two routine laboratory tests, low levels of hemoglobin and creatinine clearance are known to significantly increase the risk of death [31]. Low hemoglobin levels have been shown to be statistically significant with a prospective nonlinear association between hemoglobin concentrations and cognitive function [32]. The association between low creatinine levels and functional limitations suggests that creatinine levels are affected by factors other than renal function and muscle mass in the elderly [33]. Cognitive function also decreases in patients with slight decreases in the glomerular filtration rate [34]. Moreover, a previous study found that a group of participants who had completed at least six months of physical activity had higher hemoglobin and glomerular filtration rates when compared to a group consisting of sedentary individuals [35]. In both men and women, increased weekly amounts of either aerobic or strength exercise have been associated with significantly higher levels of creatinine [36]. Because creatinine is a metabolite of creatine decomposition, active individuals and those with large amounts of muscle mass are expected to have higher creatine conversion rates, thus leading to higher serum creatinine levels [36]. This study also found that hemoglobin and creatinine levels were higher in patients who met the recommended MVPA levels, regardless of cognitive function. In addition, TC, TG, and LDL-C levels were significantly lower, while HDL-C levels were higher in those who met the recommended MVPA levels. Research has shown that low TC and HDL-C concentrations are associated with cognitive impairment [37]. While the mechanism by which low levels of HDL-C are associated with cognitive impairment is currently unknown, HDL-C has been described as a negative risk factor for the development of cognitive impairment [38]. This may be because HDL-C slows or prevents the development of AD by preventing the aggregation and polymerization of β-amyloid [39]. As opposed to lowering TC, He et al. [40] reported that increasing HDL-C may prevent the development of cognitive impairment and dementia.

This study also had some limitations that point to suggestions for future research. First, hemoglobin levels were not separately assessed between men and women and were also expressed as means and standard deviations according to MVPA amounts. Second, kidney function was not evaluated during the assessment of creatinine. Third, physical activity amounts were solely investigated using the IPAQ. Future studies should therefore continue to investigate hemoglobin levels and glomerular filtration rates in addition to more objectively measuring physical activity levels.

## 5. Conclusions

This study shows that in elderly Korean adults, the amount of physical activity is associated with the level of cognition function. Results also indicate that increased physical activity may enhance hemoglobin and creatinine levels and improve serum lipid profiles among this group.

In sum, physical activity engagement constitutes a useful method for reducing the risk of cognitive impairment associated with aging.

## Figures and Tables

**Table 1:** The characteristics of the participants.

Variables	Male (*n* = 1348)		Female (*n* = 1398)		*p*-Value	Post-hoc
	Normality (*n* = 1232) a	Cognitive Impairment (*n* = 116) b	Normality (*n* = 1153) c	Cognitive Impairment (*n* = 245) d		
Age (years)	70.60 ± 5.03	74.50 ± 6.76	71.65 ± 5.39	74.73 ± 7.07	<0.000	a vs. b, c, d b vs. c c vs. d
Height (cm)	166.3 ± 5.69	165.8 ± 5.86	152.9 ± 5.42	165.2 ± 5.75	<0.000	a vs. c, d b vs. c, d
Weight (kg)	66.98 ± 9.32	65.50 ± 10.70	57.82 ± 8.62	56.30 ± 8.60	<0.000	a vs. c, d b vs. c, d
BMI (kg/m^2^)	24.18 ± 2.83	23.79 ± 3.43	24.72 ± 3.27	24.27 ± 3.32	<0.000	a vs. c b vs. c
WC (cm)	84.99 ± 7.65	84.00 ± 9.10	81.40 ± 8.29	81.70 ± 8.97	<0.000	a vs. c, d b vs. c
SBP (mmHg)	131.0 ± 14.5	129.6 ± 15.5	132.8 ± 15.1	133.1 ± 17.5	0.007	a vs. c
DBP (mmHg)	76.46 ± 9.28	74.38 ± 9.89	76.78 ± 9.49	76.01 ± 10.06	0.071	-

BMI, body mass index; WC, waist circumference; SBP, systolic blood pressure; DBP, diastolic blood pressure. a: Normality male, b: Cognitive impairment male, c: Normality female, d: Cognitive impairment female.

**Table 2:** Independent associations between objectively measured physical activity and cognitive function in elderly adults.

	Unadjusted		Adjusted ^a^	
	OR (95% CI)	*p*-Value	OR (95% CI)	*p*-Value
Physical activity Engaging in 150 min MVPA per week Not engaging in 150 min MVPA per week	1.00 1.63 (1.28–2.08)	<0.000	1.00 1.35 (1.05–1.74)	0.019

OR, odds ratio; CI, confidence interval; MVPA, moderate-to-vigorous physical activity; ^a^ Adjusted for age group (<75 years and ≥75 years), sex (male and female), body mass index (<25 and ≥25), and waist circumference (male, <85 cm and ≥85; female, <90 cm and ≥90).

**Table 3:** Blood variables for each group.

Variables	Normality Elderly		Cognitive Impairment Elderly		*p*-Value	Post-Hoc
	≥150 Min (*n* = 932) a	<150 Min (*n* = 1453) b	≥150 Min (*n* = 102) c	<150 Min (*n* = 259) d		
Hemoglobin (g/dL)	14.76 ± 1.30	13.74 ± 1.38	14.51 ± 1.57	13.20 ± 1.31	<0.000	a vs. b, d b vs. c, d c vs. d
Creatinine (mg/dL)	1.09 ± 0.56	0.90 ± 0.24	1.10 ± 0.26	0.93 ± 0.48	<0.000	a vs. b, d b vs. c c vs. d
TC (mg/dL)	184.8 ± 37.7	191.3 ± 40.6	168.0 ± 40.9	191.8 ± 43.6	<0.000	a vs. b, c b vs. c c vs. d
TG (mg/dL)	111.3 ± 64.2	120.7 ± 71.6	101.9 ± 54.0	117.6 ± 62.2	0.018	-
LDL-C (mg/dL)	108.8 ± 34.5	111.2 ± 36.5	95.6 ± 33.1	115.0 ± 36.8	0.002	a vs. c b vs. c c vs. d
HDL-C (mg/dL)	56.31 ± 13.3	53.58 ± 13.8	55.43 ± 12.5	53.21 ± 18.0	0.001	a vs. b

TC, total cholesterol; TG, triglycerides; LDL-C, low-density lipoprotein cholesterol; HDL-C, high-density lipoprotein cholesterol. a: ≥150 min normality, b: <150 min normality, c: ≥150 min cognitive impairment, d: <150 min cognitive impairment.

## Abbreviations

AD	Alzheimer’s disease
ACSM	American College of Sports Medicine
CDC	Centers for Disease Control and Prevention (CDC)
KDSQ-C	Korean Dementia Screening Questionnaire Cognition
IPAQ	International Physical Activity Questionnaire
MVPA	Moderate-to-vigorous physical activity
NIHMID	Nasaret International Hospital Medical Informatics Data
TC	Total cholesterol
TG	Triglycerides
HDL-C	High-density lipoprotein cholesterol
LDL-C	Low-density lipoprotein cholesterol
ORs	Odds ratios
CIs	Confidence intervals
MMSE	Mini-Mental State Examination
WHO	World Health Organization
SPSS	Statistical package for social science
